# Elderly onset intramedullary epidermoid cyst in the conus medullaris: a case report

**DOI:** 10.1186/1752-1947-9-7

**Published:** 2015-01-12

**Authors:** Takeru Ohara, Satoshi Maki, Takeo Furuya, Taigo Inada, Koshiro Kamiya, Mitsutoshi Ota, Akihiko Okawa, Osamu Ikeda, Kazuhisa Takahashi, Masashi Yamazaki, Masao Koda

**Affiliations:** 9 Department of Orthopedic Surgery, Chiba University Graduate School of Medicine, 1-8-1 Inohana, Chuo-Ku, Chiba, 2608670 Japan; 10 National Hospital Organization Chiba Medical Center, 4-1-2, Tsubakimori, Chuo-Ku, Chiba, 2608606 Japan; 11 Department of Orthopedic Surgery, Kashiwa City Hospital, 1-3 fuse, Kashiwa, Chiba, 2770825 Japan; 12 Department of Orthopedic Surgery, University of Tsukuba, 2-1-1 tenkubo, Tsukuba, Ibaraki, 3058576 Japan

**Keywords:** Epidermoid cyst, Intramedullary tumor, Conus medullaris

## Abstract

**Introduction:**

Epidermoid cysts are known as embryonic or acquired ectopic aberrations of the ectoderm. To the best of our knowledge, there are only a few reports of elderly onset intramedullary epidermoid cysts. We report a case of elderly onset intramedullary epidermoid cyst at the conus medullaris.

**Case presentation:**

A 63-year-old Japanese woman working as a farmer presented with slowly progressive gait disturbance and voiding dysfunction. A magnetic resonance imaging scan revealed an intramedullary mass lesion at L1 to L3. We diagnosed the lesion as an intramedullary spinal cord tumor. A laminectomy was performed at the level of Th12 to L3. Upon spinal cord dissection, a yellowish milky exudation erupted from the cystic lesion. We resected white cartilage-like pieces from the cystic cavity. Because the wall of the cystic lesion tightly adhered to the spinal cord parenchyma, we abandoned complete resection of the cyst wall. The pathological diagnosis was an epidermoid cyst.

**Conclusions:**

We propose that evacuation of the cyst contents is preferable, especially in cases with elderly onset and congenital origin.

**Electronic supplementary material:**

The online version of this article (doi:10.1186/1752-1947-9-7) contains supplementary material, which is available to authorized users.

## Introduction

Epidermoid cysts are known as embryonic or acquired ectopic aberrations of the ectoderm. Spinal cord epidermoid cysts are a rare condition, accounting for 1% of spinal cord tumors. In particular, intramedullary epidermoid cysts are extremely rare [1].

Past reports have described congenital intramedullary epidermoid cyst onset in children, adolescents and relatively young people. To the best of our knowledge, there are only a few reports of elderly onset intramedullary epidermoid cysts [1]. Here we report a case of elderly onset intramedullary epidermoid cyst at the conus medullaris.

## Case presentation

A 63-year-old Japanese woman working as a farmer presented with slowly progressive gait disturbance and voiding dysfunction of approximately six months’ duration. One month prior, she complained of general fatigue. She was diagnosed with chronic post renal failure caused by urinary retention. A magnetic resonance imaging (MRI) scan revealed a space-occupying lesion at the level of L1 to L3. She was subsequently referred to our institution for surgical treatment. She was medicated for mild hypertension. There was no past history of lumbar tap, myelography, spinal surgery or spinal injury.

**Figure 1:**
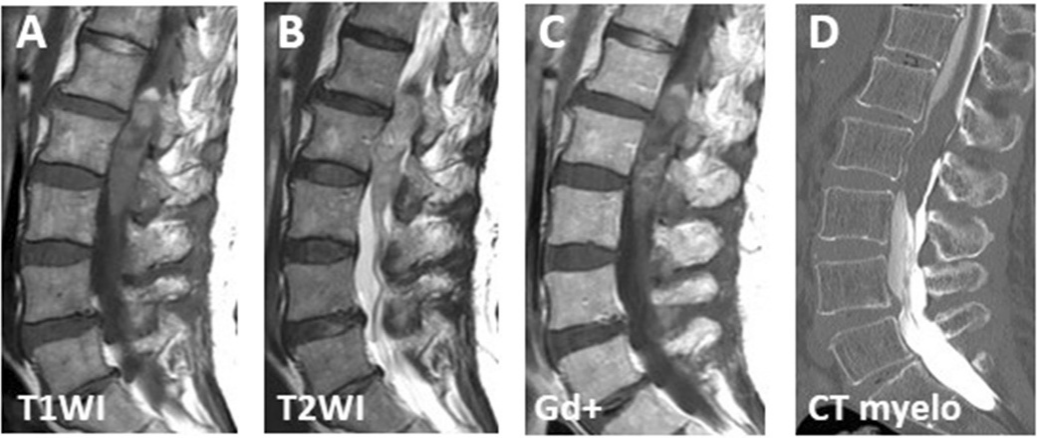
**Preoperative MRI scan: preoperative radiological findings.** An MRI scan revealed an intramedullary mass lesion extending from vertebral level L1 to L3 with mixed iso- and high-intensity change in T1-weighted image **(A)** and with isointensity change in the T2-weighted image **(B)**. Upon gadolinium enhancement, the mass lesion was heterogeneously enhanced **(C)**. A CT myelography showed a filling defect at the L1 to L3 vertebral levels **(D)**.

On admission, she could barely walk without holding on to a wall or table. Neurological examinations revealed bilateral muscular weakness (manual muscle testing one to two levels) and numbness at her distal lower extremities. A pin-prick test showed sensory disturbance in her bilateral lower legs. A deep tendon reflex test showed hypoactivity in her Achilles tendon, without pathological reflexes. Urinary catheterization was initiated because of urinary retention. She had dyschezia, with difficulty on defecation. A laboratory analysis included complete blood analysis and biochemical examination of blood on the day of admission showed no apparent abnormalities, except for elevation of creatinine and blood urea nitrogen.An MRI scan revealed an intramedullary mass lesion extending from vertebral level L1 to L3, with mixed iso- and high-intensity change in T1-weighted images, and with isointensity change in the T2-weighted image (Figure 1A, B). Upon gadolinium enhancement, the mass lesion was heterogeneously enhanced (Figure 1C). A myelography and computed tomography (CT) myelography showed a filling defect at the L1 to L3 vertebral levels (Figure 1D). Therefore, we diagnosed the lesion as an intramedullary spinal cord tumor.

**Figure 2:**
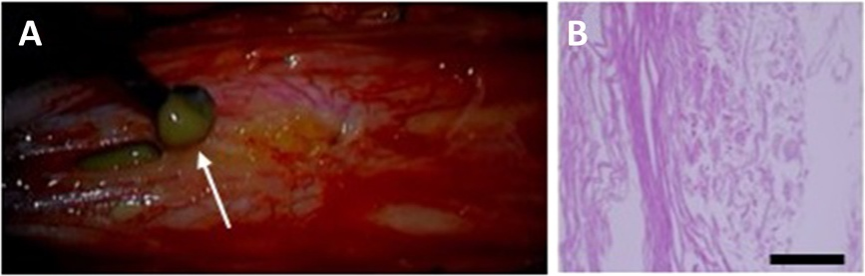
**Intraoperative and pathological findings.** After spinal cord dissection along the posterior medial sulcus, a yellowish milky exudation erupted from the cystic lesion (**A**, arrow). We resected white cartilage-like pieces from the cystic cavity (**A**, Additional file 1: Supplementary video). A photomicrograph shows pathological findings of cyst contents and capsule **(B)**. A pathological examination revealed ramified keratinized contents with a fibrous capsule **(B)**. Bar = 200μm.

She underwent surgery under general anesthesia. A laminectomy was performed at the level of Th12 to L3. Surgical microscopic observation revealed a subpial yellowish lesion with apparent spinal cord swelling. There were bifurcations of rootlets on the dorsal surface of the spinal cord at the L2 to L3 vertebral level, showing evidence of a low conus. Upon spinal cord dissection along the posterior medial sulcus, a yellowish milky exudation erupted from the cystic lesion. We resected white cartilage-like pieces from the cystic cavity (Figure 2A, Additional file 1: Supplementary video). Because the wall of the cystic lesion tightly adhered to the spinal cord parenchyma, we abandoned complete resection of the cyst wall. A pathological examination revealed ramified keratinized contents with a fibrous capsule (Figure 2B). The final pathological diagnosis was an epidermoid cyst.

The postoperative course was uneventful. Upon a six-month follow-up visit, she showed recovery of muscle weakness; she could walk without crutches. However, voiding dysfunction remained unchanged.

## Discussion

Two opposing surgical procedures for intramedullary epidermoid cysts have been reported previously. One is radical resection of the cyst wall and the other is evacuation of the cyst contents. Agarwal *et al*. reported neurological deterioration immediately after complete removal of a cervical intramedullary epidermoid cyst in a 40-year-old patient [2]. Fortunately, their patient recovered naturally, but their report suggested a risk of paralysis by complete removal of the tightly adhered cyst wall.

Most reports agree that evacuation of cyst contents is a suitable surgical procedure for intramedullary epidermoid cysts. We chose the internal decompression by evacuation of cyst contents as a surgical procedure because the cyst wall tightly adhered to the surrounding spinal cord tissue [3]. Several previous reports described that neurological recovery was obtained by the simple evacuation of cyst contents. In this context, our choice of treatment was appropriate, at least in part. Indeed, our patient showed a marked recovery of muscle power of her lower extremities. One of the possible shortcomings of cyst contents evacuation is the recurrence of neurological symptoms induced by regrowth of the cyst [4]. Tekkök *et al*. reported early recurrence of an intramedullary epidermoid cyst after evacuation of cyst contents in a pediatric patient [5]. Stevens and Schlesinger reported that one of two cases showed recurrence of an intramedullary epidermoid cyst seven years after evacuation of cyst contents in relatively young patients in their 40s [6].

Our patient showed late onset of symptoms although concomitant abnormalities, including a low conus, suggested congenital etiology. This suggests extremely low growth speed of the cyst compared with the cases in previous reports in which almost all cases were relatively young, including children and adults in their 30s and 40s. In general, surgery for spinal cord tumor in elderly patients tends to show a relatively poor outcome [7]. In contrast, surgical outcome for epidermoid cyst in elderly patients shows a satisfactory outcome as shown in previous reports and our patient [8]. This might be attributed to the low growth speed of the cyst compared with the other types of spinal cord tumors. Long-term follow-up is needed to prove this issue.

## Conclusions

We report a case of elderly onset intramedullary epidermoid cyst at the conus medullaris. We propose that evacuation of the cyst contents is preferable, especially in cases with elderly onset and congenital origin.

## Consent

Written informed consent was obtained from the patient for publication of this case report and any accompanying images. A copy of the written consent is available for review by the Editor-in-Chief of this journal.

## Electronic supplementary material

Additional file 1: **Supplementary video.** (MP4 6 MB)

## Abbreviations

CT: Computed tomography
L: Lumbar vertebra
MRI: Magnetic resonance imaging
Th: Thoracic vertebra.
